# The Different Story of *π* Bonds

**DOI:** 10.3390/molecules26133805

**Published:** 2021-06-22

**Authors:** Marco Cappelletti, Mirko Leccese, Matteo Cococcioni, Davide M. Proserpio, Rocco Martinazzo

**Affiliations:** 1 Dip. Chimica, Università degli Studi di Milano, v. Golgi 19, 20133 Milano, Italy; marco.cappelletti3@studenti.unimi.it (M.C.); mirko.leccese@unimi.it (M.L.); davide.proserpio@unimi.it (D.M.P.); 2 Dip. Fisica, Università di Pavia, via Bassi 6, 27100 Pavia, Italy; matteo.cococcioni@unipv.it; 3 Samara Center for Theoretical Materials Science (SCTMS), Samara State Technical University, 443100 Samara, Russia; 4 Institute of Molecular Science and Technologies (ISTM), Consiglio Nazionale delle Ricerche (CNR), v. Golgi 19, 20133 Milan, Italy

**Keywords:** multiple bonding, trans-bending, π distortivity, DFT, Hubbard

## Abstract

We revisit “classical” issues in multiply bonded systems between main groups elements, namely the structural distortions that may occur at the multiple bonds and that lead, e.g., to trans-bent and bond-length alternated structures. The focus is on the role that orbital hybridization and electron correlation play in this context, here analyzed with the help of simple models for σ- and π-bonds, numerically exact solutions of Hubbard Hamiltonians and *first principles* (density functional theory) investigations of an extended set of systems.

## 1. Introduction

If one asks a chemist which is the most important element of the periodic table, chances are that his choice will be carbon. Its unique properties make carbon dominant not only in its very own branch of chemistry, but in virtually all the other fields. Together with its incredible versatility, with it being able to form all sorts of structures from simple molecules to proteins, and from 0D to 3D materials, carbon compounds are also capable of displaying extraordinary transport properties [1,2]. The other group 14 congeners are also of great importance in today’s science and technology—thanks to their valuable semiconducting properties—but none of them has been as “successful” as carbon, despite the expected similarities in chemistry. The differences between carbon and even its closest congener are evident: Silicon on earth is practically only found in the form of silicate [3] and it is not even close to the richness of carbon chemistry. Moreover, despite it being the second most abundant element on the earth’s crust (21.22% atomic percent compared to 1.94% for carbon) [3], Si does not play a pivotal role in life [4]. As a matter of fact, very few bio-molecules as well as biochemical processes involve the silicon atom [4], contrary to what happens in other second-row elements such as phosphorus and sulfur.

The main feature of carbon is to form strong multiple bonds with both itself and other first-row elements, an attitude that strikingly contrasts with the tendency of its congeners to preferentially form three-dimensional $sp^{3}$ structures [5] and to be quite reluctant concerning π bonds. Several simple X${}_{2}$Y${}_{4}$ molecules (X = group 14 element, Y = substituent) prefer an unusual, “double bridged” structure rather than forming a double bond between the two Xs, a tendency that becomes increasingly evident when moving down the group [6]. Most often, double bonds in pseudo-olefins and derivatives are found to be weaker than a single bond (e.g., the energy needed to break H${}_{2}$SiSiH${}_{2}$ into two SiH${}_{2}$ fragments, ≃60 kcal mol${}^{-1}$, is much smaller than the average Si-Si bond energy, ≃95 kcal mol${}^{-1}$), consistently with the Carter–Goddard theory [7].

In spite of these oddities, several structures containing double [8,9] and triple [10,11] bonds between higher group 14 elements—as well as trisillalenes [12], and exotic aromatic [13] and antiaromatic [14] rings—have been realized and the celebrated “double bond rule” disproved. As a matter of fact, almost all group 13–16 elements are nowadays known to be able to form doubly bonded species [15,16]. This provides a zoo of structures that help to understand multiple bonding beyond the biased view provided by carbon chemistry. At the same time, this allows us to improve our understanding of π-bonding in carbon compounds (by freeing it from misconceptions) and provides new opportunities for applications. Si-Si double bonds, for instance, have been shown to feature rather unique structures [17] (with no analogue in carbon chemistry) that are currently being investigated for integration in carbon π-electronic systems [18]. Si monolayers (a.k.a. silicene)—recently identified in a quasi free-standing form [19], in full analogy with graphene [20,21,22,23]—are known to harbor a rather rich phase diagram as a function of external electric and magnetic fields, displaying both a normal band insulator phase and more exotic quantum anomalous (or spin Hall insulator), valley polarized metal and spin-valley polarized metal phases [24].

One striking feature of the above-mentioned low-coordinated structures is that they are far from being “$sp^{2}$”. They typically appear distorted at the multiple bonds, which is known as trans-bending in silenes, disilenes and higher group analogues and buckling in silicene [25] and germanene [26]. This has lead over the years to several explanations, ranging from σ−π mixing and coordinate-dative bonds between (singlet) pseudo-carbenes in molecules [27,28,29,30,31] up to π capability [32] and pseudo Jahn–Teller distortion [33,34] in one- and two-dimensional systems. In a recent paper [35] one of the present authors introduced a unified description of multiple bonding on the basis of an accurate, yet “classical” σ+π model, which applies equally well to finite-sized and extended systems and accounts, at least qualitatively, for the effects of electronegative and/or π donating substituents. The model allows one to single out the main ingredients governing such angular distortion and makes evident that the equilibrium molecular structure is determined by a delicate interplay between a distorting σ force and a resisting π one. Furthermore, the difference between carbon and silicon was shown to be due to atomic-like properties, albeit in a counter-intuitive way: It is the larger interaction between valence orbitals in C—which in turns arises from the similar size of its s and p valence shells—that determines a larger π bending stiffness. This occurs because a destructive interference between s and p orbitals arises when forming a distorted π bond and establishes a direct link between the unusual atomic properties of second-row elements (related to the presence of poorly screened *p* valence orbitals [36,37]) and their unique chemistry.

The idea that the σ and the π electrons act oppositely in determining the equilibrium structure has a long history. In the case of trans-bending discussed above the concept was introduced almost 20 years ago by Danovich et al. [38] for HC≡SiH and HSi≡SiH—and later generalized to several triply bonded binary systems [39]—but its origin is even more remote in connection with the bond alternating distortion in conjugated and aromatic molecules [40], where it dates back to early observations by Longuet-Higgins and Salem of 1959 [41]. Since then, extensive theoretical and experimental investigations have been performed and convincing evidence has accumulated showing that π-bonds are always distortive w.r.t. bond alternation [40,42,43,44], a rather odd result in light of the widespread belief that electron delocalization in aromatic systems is the key for a uniform or quasi-uniform bond length pattern. In fact, it is only the presence of a σ resistance that makes bond-equalized structures like benzene possible, and that makes the distortivity of π-bonds silent [40,42,43,44]. More generally, such a distortive nature of π-bonds can be traced back to Peierl’s observation that a one-dimensional crystal with a half-filled *s*-like band is unstable against dimerization (i.e., bond-length alternation) since the ensuing band folding lowers all the occupied energy levels [45]. In a sense, Shaik, Hiberty and coworkers [40,42,43,44] generalized these findings and proved convincingly that such distortivity does matter in finite (and small) systems as well, and coexists in harmony with aromaticity: The difference between aromaticity and antiaromaticity is seen in different resonance and excitation energies; both systems, however, display a distortive tendency [40]. Again, this aspect is not merely academic: Peierls’ distortion in an extended system produces periodic fluctuations in the electron density, known as charge density waves, that are an example of collective charge transport. These are potentially relevant for carbon systems too, given that all-carbon linear chains (a.k.a. carbynes) of ever increasing length are currently being reported [46,47] and the ultimate 1D carbon material is within reach in the near future.

The above discussion makes clear that the picture of π bonds that results from carbon chemistry is—at the least—rather limited and a different story of multiple bonds has to be taught to the next generation of chemists: π and σ bonds act oppositely in determining the equilibrium structure (either distortive or resisting depending on the kind of distortion considered) and it is only their interplay that determines the final structure attained by the multiply bonded system. Importantly, electron correlation plays a pivotal role in this context, since π bonds are far from the molecular orbital limit and their stiffness/distortivity can be tuned to a large extent by changing the effective repulsion between π electrons, e.g., by means of π-donating/accepting substituents or physical charge doping.

In this manuscript we address some aspects concerning this non-orthodox but more appropriate picture of multiple bonds, with the help of both first principles calculations and model results. After introducing in Section 2 the methods used, in Section 3 we take a closer look at the role that substituents play in determining the angular distortions at Si-Si double bonds and then, in Section 4, we discuss bond length alternation and π distortivity in cyclic carbon systems of variable size. Finally, we summarize and conclude in Section 5.

## 2. Methods

First principle results presented below were obtained with Density Functional Theory (DFT). All-electron density functional theory calculations were performed using several density functionals (namely, the popular B3LYP hybrid functional and the Minnesota’s family of meta-hybrid functionals [48]), in conjunction with the Pople’s 6-31++G${}^{**}$ atomic-orbital basis-set used to expand the Kohn–Sham orbitals, in a spin-unrestricted framework. Calculations were performed with the Gaussian16 code [49] using tight convergence criteria for both the electronic and the geometric optimizations. The converged electronic structures were analyzed with the help of the powerful natural bond orbital analysis [50,51,52] and the stability of the optimized molecular geometries (See Supplementary Material) was always checked with a frequency analysis. Generalized internal coordinates were employed to perform the constrained geometry optimizations. Excited state calculations were performed following Casida’s strategy in the framework of linear-response time-dependent DFT, as implemented in Gaussian16, and employing the same functionals above in the adiabatic approximation.

In addition to first principle calculations we made profitable use of some simple modeling, along the lines of reference [35] that vividly express Pauli’s teachings on chemical bonding. There we assessed the reliability of a “classical” σ+π bonding scheme in describing the chemical bond in simple Y${}_{2}$X = XY${}_{2}$ systems (Y = substituent and X = group 14 element) assuming that the X atoms form one π-like bond and three non-bent σ bonds using hybrid orbitals $sp^{\tau }$ and $sp^{\mu }$ (for X-Y and X-X, respectively). The hybridization indexes τ and μ were then determined by the spatial arrangement of the ligands around each X atom, following Coulson’s directionality theorem, and dictated, in turn, the nature of the atomic states involved in π-bonding, i.e., the hybridization index λ of the $sp^{\lambda }$ hybrids used in such a bond. Bonding was described as independently provided by σ- and π-like correlated contributions which were obtained, in turn, from a two-electron two-state Hubbard model. The latter is a simple (analytically solvable) correlated model in which binding is determined by the competition between a hopping energy −t (where t>0 for an *s*-like bond) and the “on-site” Coulomb energy *U*. For an accurate modeling orbital overlap was taken into account and the effective hoppings between the hybrids involved in the σ- and π-like bonds were expressed in terms of atomic orbital (AO) contributions, using the Slater–Koster parameters [53] of references [54,55]. Likewise, bare on-site Coulomb energy was approximately expressed in terms of AO contributions and hybridization indexes, and screening was described by AO contributions and derived from available results [56] and from the triplet–singlet separation energies in methylene and sylilene.

The model can be generalized to a large extent, again in the spirit of Pauling’s vision of bonding, to address more complicated systems. Firstly, the localized σ bonds can always be taken to involve two electrons at a time, using hybrids appropriate for each atom neighborhood. As described in reference [35] (SI) for three σ bonds directed along ${\hat{n}}_{1},{\hat{n}}_{2}$ and ${\hat{n}}_{3}$, the corresponding indexes $\tau_{i}$ follow as $\tau_{i}=-{\hat{n}}_{j}{\hat{n}}_{k}/ [\left({\hat{n}}_{i}{\hat{n}}_{j}\right)\left({\hat{n}}_{i}{\hat{n}}_{k}\right)]$ (here i,j,k=1,2,3, i≠j≠k) and fix the hybridization index of the fourth hybrid $sp^{\lambda }$ that can be formed out of the s,p AOs. The latter hybrid is the one involved in the π-bond and its index, easily obtained by exploiting conservation of the *s* (or *p*) weights upon hybridization, reads as $\lambda ={\left[1-\sum_{i}{\left(1+\tau_{i}\right)}^{-1}\right]}^{-1}-1$ (see SI of reference [35]). This establishes the general connection between the geometry of the σ backbone and the nature of the effective orbitals forming the π bonds. Secondly, π electrons, which are typically much more correlated than σ ones, can be investigated numerically using the Hubbard Hamiltonian which, for orthogonal atomic states, reads (in second quantized form) as

$$H=-\sum_{ij}\sum_{\sigma }t_{ij}a_{i,\sigma }^{†}a_{j,\sigma }+\sum_{i}U_{i}n_{i,↑}n_{i,↓}$$

Here, the first sum runs over all relevant pairs and spin (σ=↑,↓), $t_{ij}$ is the energy associated with an electron hopping from site *j* to site *i* (typically limited to nearest neighbors), the second sum runs over the π sites, $n_{i,\sigma }=a_{i,\sigma }^{†}a_{i,\sigma }$ is a number operator and $U_{i}$ is the on-site interaction strength ($U_{i}\equiv U$ for equivalent sites). The model can be exactly solved for a reasonable number of electrons (n≈16), using appropriate parameters dictated by “the chemistry”, and can thus provide valuable insights into π bonds delocalized over a number of sites. To this end we employed the HΦ program package [57] that allows exact diagonalization for small sized systems and (yet numerically exact) iterative Lanczos diagonalization for larger ones.

We stress here that the aim of the just outlined modeling is not, of course, to replace the accurate description provided by first principles means, but rather is to complement the results of such accurate calculations by providing insights into the electronic structure and singling out the main factors affecting the physical behavior.

## 3. Trans-Bending

As discussed at length in reference [35], when applying the simple model outlined above to trans-bending in pseudo-olefines few key ingredients governing such angular distortion become apparent: (i) A σ−π separation is yet relevant for distorted bonds, and a classical picture consisting of two inequivalent bonds (one σ and one π-like) is adequate in most situations; (ii) the equilibrium molecular structure is determined by a delicate interplay between a distorting σ force and a resisting π one; and (iii) electron correlation plays a subtle role in that it tunes the strength of the π bond and its bending stiffness.

**Figure 1:**
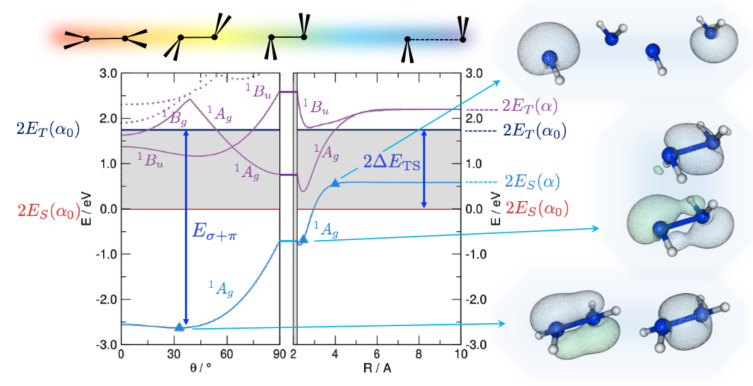
DFT energetics of disilene (H${}_{2}$SiSiH${}_{2}$) along the path devised by Trinquier and Malrieu [27], sketched on top of the graphs, along with some important energy values: the triplet-singlet separation ($\Delta E_{TS}$) of the fragments and the strength of the double bond ($E_{\sigma +\pi }$). Energy is referenced to the pair of singlet fragments in their equilibrium geometry ($\alpha_{0}$). Shown also are the lowest lying singlet excited states (solid and dotted lines in purple), and the relevant (occupied) Natural Bonding Orbitals for selected structures along the path, as indicated.

That the “classical” σ+π bonding is appropriate in most situation is not obvious since, as suggested long ago by Trinquier and Malrieu [27,28,29], an alternative and equally reasonable bonding scheme involving n→p bonds becomes possible when the fragments have a singlet ground state (which is typically the case for silylenes and higher main group analogues). The latter bonding scheme is particularly appealing in this context since, with the maximum n→p bond strength at $\theta ∼45^{\circ }$, it naturally predicts a trans-bent geometry. The situation is here analyzed with first principle calculations and is exemplified in Figure 1, reporting the disilene (H${}_{2}$SiSiH${}_{2}$) energetics along the path devised by Trinquier and Malrieu [27] to investigate pyramidalization. From right to left, the two silylenes are first brought to the equilibrium distance in an orthogonal bent geometry (Step 1); then—while keeping a $C_{2h}$ symmetry—they recover planarity through trans-bending (Step 2). In Step 1 the energy profile is rather flat when the fragments are in the singlet state (which is the ground-state for SiH${}_{2}$) because two empty p orbitals face each other, while only a pp(σ) interaction is allowed when they are in the triplet (here the first excited state). The latter involves only the two p orbitals orthogonal to the HSiH planes, the remaining two *p* electrons being left unpaired. Upon bending, the “triplet–triplet” state becomes strongly interacting because of pairing (and orbital rearrangement) of the latter two *e*, while the “singlet–singlet” one becomes attractive because of n→p bonding.

In Figure 1 we show the results of constrained optimizations performed at the B3LYP level of theory. Displayed there are the energies of both the ground, “singlet–singlet” state dissociating into a pair of singlets and the first singlet excited state, which is the “triplet–triplet” state appropriate for σ+π binding. We kept frozen the angle ∠HSiH at its equilibrium value since in this way the hybrids involved in the HSi bonds remain unchanged upon bending and the strength of such bonds does not affect the overall energetics. Notice that results are referenced to the energy of a pair of singlets at their equilibrium geometry (in particular, the equilibrium bond angle $\alpha_{0}$); this is why the ground-state curve has a non-zero asymptotic value. The results displayed in Figure 1 show that the ground-state interaction in Step 1 is attractive, thereby ruling out the possibility that it keeps its “singlet–singlet” character. Rather, an avoided crossing with the “triplet–triplet” state occurs somewhere between R∼4 Å and the equilibrium distance $R_{e}∼2$ Å and allows the pp(σ) bond to show up in the ground-state energetics. This is confirmed by the Natural Bond Orbital (NBO) analysis which indeed shows a change of character in the ground state electronic structure: A pair of (symmetry equivalent) n→p bonds is appropriate at large Si-Si distances, but at closer separation two distinct bonds, a σ bond and a distorted π one, clearly emerge. Trans-bending stabilizes further the binding in the ground-state and leads to a final distorted equilibrium structure which presents a σ and a distorted π bond. Interestingly, the same happens in the ${}^{1}B_{u}$ electronic state, where the odd symmetry with respect to inversion implies some antibonding character in the π bond. This state is indeed analogous to the ${}^{1}B_{u}$ state of ethylene (${}^{1}B_{1u}$ in $D_{2h}$ symmetry) which is known to be of $\pi \pi^{*}$ type. Thus, it presents a reduced resistance to bending, as manifested by the deeper minimum in the potential energy curve (violet line in Figure 1). Similar results have been obtained for several substituted disilines, and compared in detail to C analogues in reference [35]. It has been further shown that, as a consequence of the delicate interplay between σ-distorting and π-restoring forces, pyramidalization diminishes and disappears when compressing the bond, while it increases upon stretching. Ethylene and its derivatives, if stretched enough, do undergo pyramidilazation and, on the other hand, disilenes flatten when properly compressed [35].

The model of reference [35] further accounts, at least qualitatively, for the effects of the substituents at the double bond. According to Bent’s rule, the electronegativity of Y affects the bending angle ∠YXY (the larger the electronegativity the smaller the angle is); hence the hybridization index of the hybrids involved in the σ- and the π-like bonds. In this way, Y’s electronegativity is seen to increase the tendency to distortion, because of an increased distortion force exerted by the σ bond and, to a lesser extent, a reduced resistance of the π one. Likewise, since π-donating (π-acceptor) species are expected to increase (decrease) the role of the Coulomb repulsion in the π bond, they reduce (increase) the π resistance to distortion.

**Figure 2:**
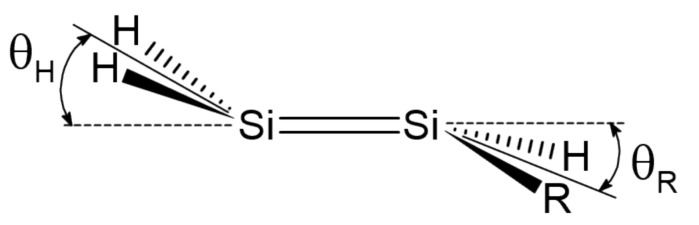
Pyramidalization angles $\theta_{H}$ and $\theta_{R}$ in mono-substituted disilenes.

**Figure 3:**
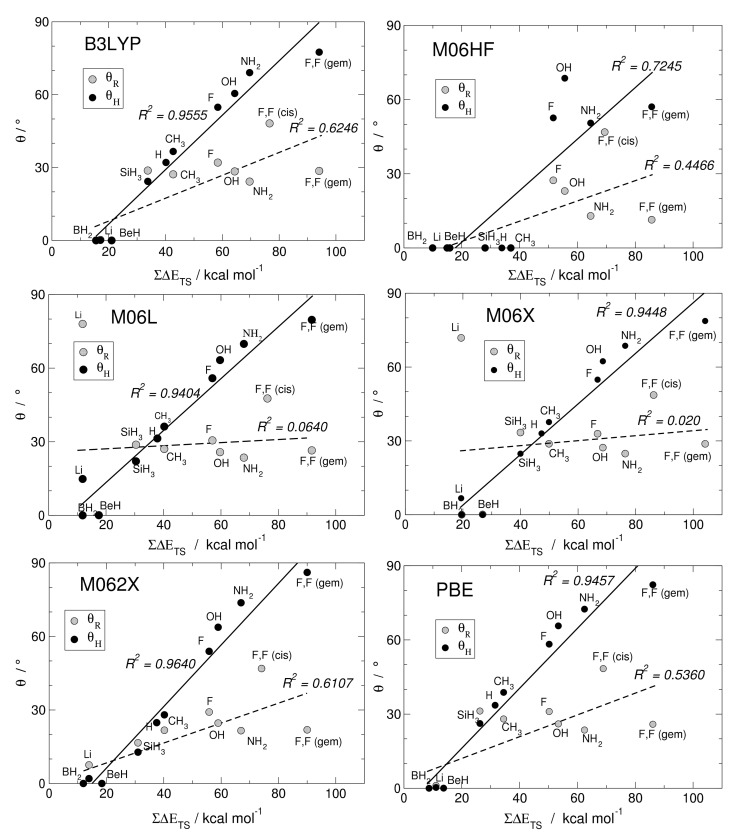
Angles of pyramidalization vs. $\sum \Delta E_{TS}$ of different mono- and di-substituted disilenes computed with B3LYP, M06HF, M06L, M06X, M062X, PBE functionals, along with their linear regressions and the corresponding (squared) correlation coefficients.

**Figure 4:**
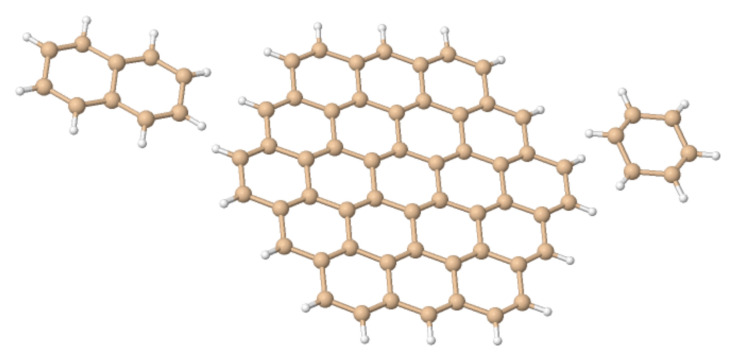
The “silicene flakes” considered in Table 1. From left to right, Si${}_{10}$–naphatelene, Si${}_{54}$–circumcoronene and hexasilabenzene.

Here, we investigate in detail substituent effects with first principle means following the original work by Karni and Apeloig on mono-substituted disilenes [58]. Calculated values of pyramidalization angles $\theta_{H}$ and $\theta_{R}$ (see Figure 2 for their definition) of H_2_Si=SiHR disilenes with R=Li, BeH, BH_2_, CH_3_, SiH_3_, NH_2_, OH and F are plotted in Figure 3 as functions of the sum of triplet–singlet energy difference, $\sum \;\Delta E_{TS}$, of the building fragments, i.e., SiH${}_{2}$ and SiHR, at different level of theory (density functional); also shown in the same graphs are the results for H_2_Si=SiF_2_ and FHSi=SiHF. In general, all the functionals considered agree well with each other (and with the original MP3/MP4 results by Karni and Apeloig [58]), except M06HF. The latter tends to underestimate the disilene pyramidalization, and predicts many disilenes to be planar. This makes clear that electron correlation plays a major role in angular distortions, as already observed in reference [35] for several systems. Table 1 shows for instance the buckling height computed for different “silicene flakes” or “Si dots” (see Figure 4) with the same functionals above, including bare HF results which are seen to severely underestimate pyramidalization.

In Figure 3 there are few further oddities (e.g., M06L and M06 dramatically fail in the R = Li case, probably because of the small HF exchange they include), and overall B3LYP and M062X turn out to perform better than the others when the comparison is made with reference [58]. Henceforth, we can focus on the latter two cases. The general trend of $\theta_{H}$ and $\theta_{R}$ in terms of different substituents is evident: The more electronegative and π-donating R is, the more pyramidalized the disilene. When R = NH_2_, OH and F, in fact, the largest distortions are induced in the mono-substituted case; on the other hand, the electropositive and π-accepting Li, BeH and BH_2_ substituents drastically reduce the pyramidalization angles. Out of the two effects, π-donation or acceptation abilities seems to most affect distortions: Pyramidalization of the unsubstituted silicon increases in the F, OH, NH_2_ series, the latter inducing the highest pyramidalization angles in mono-substituted disilenes ($\theta_{H}=69^{\circ }$ and $74^{\circ }$ according to B3LYP and M062X, respectively), whereas when R=BeH and BH_2_ disilenes turn out to be flat. It is thus clear that electron density in the $\pi^{*}$ MO orbital (or π on-site repulsion in the Hubbard bond description) is particularly effective on distortion. This is confirmed by the fact that the substituted silicon decreases its distortion in the said series, as $n\to \pi^{*}$ interactions give rise to a partial negative charge in the other silicon, while the substituted one is less affected. Here Hydrogen, being slightly more electronegative than silicon, should be considered mildly distortive. As a matter of fact, disilene (H_2_Si=SiH_2_) possess higher pyramidalization angles than H_2_Si=SiH(SiH_3_). In the doubly substituted disilene H_2_Si=SiF_2_ the distortion induced to the unsubstituted silicon is even larger, the pyramidalization angle reaching the largest value of $86^{\circ }$, at the M062X level of theory.

The linear correlation between pyramidalization angles and the sum of the triplet–singlet energy differences uncovered by Karni and Apeloig in reference [58] is excellent in the case of $\theta_{H}$, while for the substituted silicon seem to be a little farfetched. The computed triplet–singlet energy differences show that in most cases silylenes possess a singlet ground state, making them stable on their own and less prone to forming a bond. As a matter of fact, the only substituents which induce negative values of $\Delta E_{TS}$ are the same that cause disilenes to flatten. This is somewhat in agreement with the Malrieu and Trinquier’s rule for predicting trans-bending (i.e., $\sum \Delta E_{TS}\geq E_{BE}$) [27,28,29], especially considering that, by contrast, carbenes have an energetically favored triplet state and form stable, flat ethylenes. However, distortive substituents can yet induce a singlet ground state, with $\Delta E_{TS}$ comparable to silylenes. In tetraazafulvalenes (electron-rich olefines with four N substituents), the large triplet–singlet splitting makes the binding energy very small (few kcal mol${}^{-1}$), although still enough to make some species isolable and characterizable [59]. In F_2_C=CF_2_ the binding energy $E_{BE}$ is smaller than the triplet–singlet energy difference ($E_{BE}$ = 75.0 kcal mol^−1^ vs. $\sum \;\Delta E_{TS}$ = 142.5 according to M062X), but the molecule is found to be planar, at odds with the rule. In fact, in reference [35] it was argued that the correlation between pyramidalization and $\sum \;\Delta E_{TS}$ is somewhat incidental: The same factors favoring distortion through σ-strengthening/π-weakening determine an increase of the triplet–singlet separation. For instance, electronegative substituents stabilize the *n*-like state in SiY${}_{2}$, while they do not affect the *p*-like one, and hence increase $\Delta E_{TS}$. Likewise, π-donors destabilize the triplet state by introducing Coulomb repulsion in the *p*-like orbital.

The effects of highly distortive substituents are also reflected in the stretching of the Si=Si bond. The computed bond lengths in mono- and di-substituted disilenes are seen to increase as $\sum \;\Delta E_{TS}$ increases (here not reported). In this case too, the π-donation ability affects the double bond much more than electronegativity, remarkably for the F, OH, NH_2_ series. However, no trend appears in less distortive substituents. As a matter of fact, the computed bond lengths turn out to be very similar in both flat and considerably pyramidalized disilenes. M062X even predicts that the shortest Si=Si bond is in H_2_Si=SiH(SiH_3_), for which $\theta_{H}=17^{\circ }$ and $\theta_{R}=13^{\circ }$, rather than in a flat disilene. Thus, a correlation between angular distortion and bond length stretching cannot be safely assumed.

In their paper, Karni and Apeloig found further a dramatic weakening of the bond in heavily distorted disilenes. Extrapolating the results, they predicted that disilenes for which $\sum \;\Delta E_{ST}$ is greater than 120 kcal/mol would spontaneously dissociate in two fragments. However, as pointed out by Carter and Goddard [7], such decreasing in binding energies is mainly due to the increase in $\sum \;\Delta E_{ST}$ rather than an actual weakening of the bond. In fact, in reference [35] a roughly constant (if not increasing) trend was found when considering $E_{\sigma +\pi }$ (the binding energy with respect to the triplet–triplet state). This proves that it is not a proper weakening of the bond, but rather a growing stabilization of singlet state silylenes. The same conclusion can be deduced by observing that heavier congeners of silicon typically display smaller M=M binding energies compared to the single bonded dimetallane M-M [60].

As a final remark we notice that when considering highly distorted structures the distortion may be so great that different structural isomers become more stable. The most important disilene isomers are found to be the single bonded R_3_SiSiR isomer and the double bridged RSi($\eta_{2}$-R_2_)SiR structure. For instance, at the M062X level of theory we find that in tetra-substituted disilenes Si${}_{2}$R${}_{4}$ the energy of the single-bonded (double bridge) structure—referenced to the double bonded one—is −12.90 (−15.13), −11.50 (−11.96) and −10.51 (−6.85) kcal mol${}^{-1}$ for R = NH${}_{2}$, OH and F, respectively.

## 4. Bond-Length Alternation

Next we consider a different structural distortion, namely that occurring in the length of the double bonds, using two kinds of target systems. In a first investigation we addressed energetics and distortion in *n*-annulenes—cyclic molecules of general formula C${}_{n}$H${}_{n}$—with first principle means; in the second one, we applied numerically exact diagonalization techniques to π Hubbard models of cyclic molecules.

### 4.1. n-Annulenes

**Figure 5:**
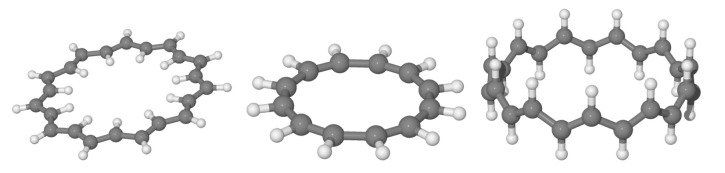
The anti-, cis- and trans-annulene structures considered in this work.

We investigated the prototypical example of molecules to which Huckel theory applies, in the different structural variants depicted schematically in Figure 5 and for variable size. For each *n* we optimized two planar structures, the anti and the cis one, under constrains of a flat geometry and managed to obtain an all-trans structure for n≥8. The three variants considered present different delocalized bonds between carbon *p* electrons and allow one to probe the role of the σ skeleton separately from that of the delocalized bond. The first two kinds of structures feature an exact σ−π separation and p−p interactions that are not affected by the ring size. Rather it is their different σ skeleton that determines their relative stability, with the cis structure preferred for small *n* (e.g., cyclobutadiene, benzene, etc.) and the anti structure favored for large *n*. The trans sequence, on the other hand, features an ideal “environment” for σ bonding, only marginally affected by the ring size, and a (cis-bent) interaction between *p* orbitals for π bonding that becomes increasingly important when increasing *n*. Notice that the three structures have two different limits for n→∞: One is rather odd (the one attained by the cis sequence), the other is the stable configuration of trans-polyacetilene, here reached from two different “directions”. This infinite size limit allows one to access properties of the extended system trans-polyacetilene from a molecular perspective—free of finite-size effects thanks to the ring topology—and to apply theory levels higher than those typically available in the condensed phase. Here, we used several density functionals, as described in the Section 2, but present the most reliable results only, which were obtained with M062X.

**Figure 6:**
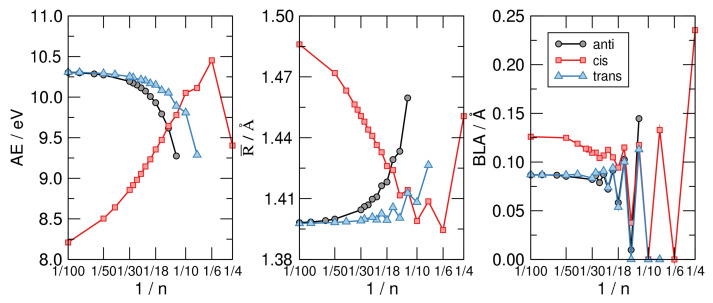
From left to right: The atomization energy per C atom (AE), the average CC bond length ($\bar{R}$) and the bond length alternation (BLA) for the C${}_{n}$H${}_{n}$ structures exemplified in Figure 5, as functions of 1/n, on a linear-log scale.

Figure 6 shows the main results of our investigation. The left panel displays the atomization energy per C atom—i.e., the energy of the reaction $\frac{1}{n}$C${}_{n}$H${}_{n}$→ C + H—as a function of 1/n in a linear-log scale. The graph makes clear the stability order mentioned above and the infinite-size limiting behavior alluded to, where the anti and trans sequence tend to the same common value AE∼10.3 eV which is our computed AE for trans-polyacetilene. The latter is about 2 eV higher than the hypothetical chain with CH bonds made with pure C *p* orbitals, and C-C σ bonds built with sp hydrids. In a sense, it is a carbyne-like system where for any structural unit C${}_{2}$ a π bond is replaced with two (odd) CH bonds.

The middle panel of Figure 6 highlights the structural differences between the isomers considered and shows at the same time that the most stable structure shares a common averaged CC bond length, disregarding the evident alternation due to the aromaticity–antiaromaticity for small *n*. The latter is more evident in the right panel of the same figure, where the bond-length alternation is seen to undergo wild oscillatory behavior which extends up to n∼ 30. From the latter graph we see that the distortivity is highly modulated by the aromatic–antiaromatic character of the π cloud, anti-aromatic molecules being highly distortive except for the smallest trans-structure optimized (n=8) where the poor overlap between *p* orbital gives to the π cloud a little weight on the overall energetics. Apart from this, the BLA vanishes (or is vanishingly small) up to n=10 for the cis-sequence and up to n=14 for the other two sequences, a manifestation of the delicate interplay between the π distortivity and the σ resistivity. The case n=4 (obtained only in the flat, cis-configuration) seems to be a bit off the general trend, featuring a much larger BLA and average bond length $\bar{R}$. This is probably a consequence of the ring strain which is considered to be the major effect causing distortion in this system, as also confirmed by the energetics shown in the left panel of Figure 6.

**Figure 7:**
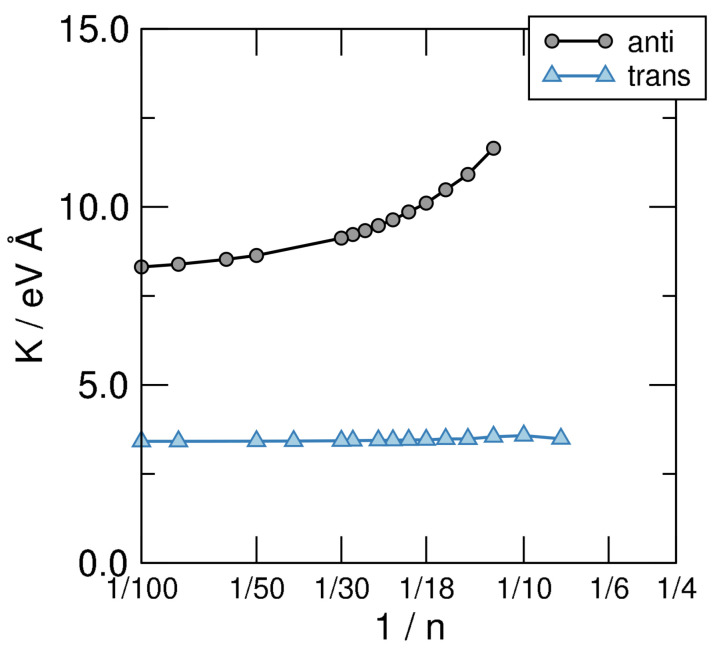
Bending stiffness of the structural sequences defined in Figure 5, on a linear-log scale.

As mentioned above, the results obtained for the different sequences of annulenes allow one to extrapolate molecular properties to the extended system. Of particular interest in this context is the bending stiffness which can be defined, for a rod of length *L*, as the second derivative of its energy density w.r.t. the curvature κ, or equivalently, for our C${}_{n}$H${}_{n}$ molecules, as

$$K=\frac{1}{\bar{\rho }}\frac{\partial^{2}\epsilon }{\partial \kappa^{2}}$$

where ϵ=E/n≡−AE is the formation energy per structural unit (provided *E* is referenced to the atomized limit), $\bar{\rho }$ is the average bond length projected onto the molecular axis and $\kappa^{-1}$ is the ring radius. Since in the limit we are interested in $2\pi \kappa^{-1}\approx n\bar{\rho }$ the bending stiffness takes the form

$$K=\lim_{n\to \infty }\frac{\bar{\rho }}{4\pi^{2}}\frac{\partial^{2}\epsilon }{\partial {\left(1/n\right)}^{2}}$$

which can be used to obtain *K* by fitting the AE vs. 1/n curves shown in the left panel of Figure 6 to a smooth curve and taking its second derivative w.r.t. 1/n. The results of such a calculation are shown in Figure 7, where the structures investigated in this manuscript are seen to attain clearly different limits as n→∞. Disregarding the unphysical limit of the cis-sequence (which is unstable w.r.t. the bending deformation since K<0, as evident from the left panel of Figure 6) the anti and trans ones present rather different values of *K*, namely K∼8.3 eV Å for the first and K∼3.3 eV Å for the second. The latter two give the stiffness of trans-polyacetilene for bending in two different ways: While the limiting anti-structure describes an in-plane deformation of the “ribbon”, the trans- sequence mimics an out-of-plane deformation. In turn, the first one involves the σ backbone only—the bending of the σ bonds between C atoms—while the second calls into question the π bonds only. The latter is thus a manifestation of the π-resistivity to cis-bending which, in turn, in full analogy with what is described in Section 3, could be expressed in terms of hopping energy between AOs and effective interaction terms. This shows how atomic properties show up in the extended system and determine the properties of the material. It is worth noting that the computed value of the π-bending stiffness compares remarkably well with the value of *K* estimated for carbynes (K=3.56 eV Å) [61], as expected since the two structures involve rather similar bonds in such bending deformation.

### 4.2. π-Distortivity from Hubbard Calculations

The results of Figure 6 clearly show that π distortivity is operative in annulenes, and that only the presence of “aromaticiy” (for small *n*) can reduce it to the extent that BLA disappears. To investigate this issue further and unequivocally assess the role of π distortivity in determining the equilibrium structure we considered the Hubbard Hamiltonian described in Section 2 as a model for the π electrons in annulenes. This is complementary to the original ab initio analysis by Hiberty et al. [43] and Shaik et al. [44] in that it offers the unique possibility of ascertaining and quantifying the π distortivity in the absence of the opposing σ resistivity which is unavoidably present in real systems.

To this end, we considered cyclic lattices with *n* = 4–14 sites at half-filling (i.e., with *n* electrons) and computed their energies in the nearest-neighbor approximation where only hopping between nearest neighbors is allowed. The fully delocalized state has been represented by *n* equivalent hopping parameters *t* between the adjacent sites, and the bond length alternation was simulated by alternating $t_{+}>t_{-}$ along the ring. In order to avoid contrived energy losses or gains, we set $t_{+}+t_{-}=2t$ (see also the discussion below on this issue) and used $\delta =\Delta t/t=(t_{+}-t_{-})/t$ as BLA coordinate ($t_{\pm }=t\;\left(1\pm \delta /2\right)$). The latter was varied under variable interaction strength *U* from the delocalized situation (δ=0) to the fully localized one featuring n/2 non-interacting dimers (δ=2). The U/t=0 case is the molecular orbital limit where Huckel’s theory applies and where a marked distinction between aromatic and antiaromatic systems is evident; this is also the limit where Peierl’s argument applies and where distortivity has to be expected, at least for large *n*. The key issue is, of course, to what extent electron interaction affects these results.

**Figure 8:**
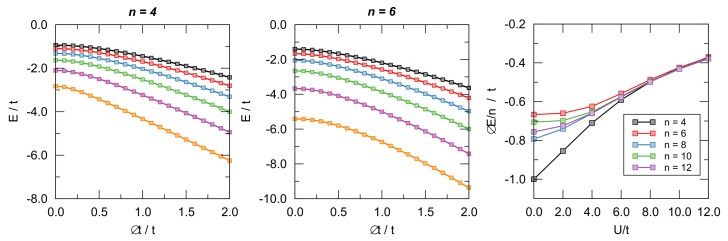
**Left** and **central** panel: Ground-state energy of the n=4,6 Hubbard models for different values of U/t as functions of Δt/t. From **bottom** to **top** U/t=2,4,6,…. **Right** panel: distortion energy $E_{\delta =2}-E_{\delta =0}$ per site as a function of U/t for different number of sites.

Representative examples of the computed ground-state energies as functions of δ are provided in Figure 8 for n=4,6 and different values of on-site energy *U*, as indicated. The results are rather clear: The π-electrons are always distortive, and the most stable arrangement is obtained for δ=2, i.e., when the chain is fully dimerized. The same figure also makes apparent the differences between aromatic and antiaromatic systems, see in particular the right panel where the “distortion energy” per site $\Delta E/n=(E_{\delta =2}-E_{\delta =0})/n$ is plotted as a function of U/t for several *n*’s. The aromatic-antiaromatic alternation is evident, with antiaromatic systems more distortive than aromatic ones, although the differences flatten out as either *n* or U/t increases.

The above findings call for a more rigorous analysis of the effect that interaction and molecular size have on the distortive character of the π system. To this end, we regarded the *n*-site lattice Hamiltonian as a function of its *n* hoppings, $H=H\left(t_{1},t_{2},\ldots ,t_{n}\right)\equiv H\left(t\right)$, and looked at the ground-state energy Hessian

$$H_{ij}=\frac{\partial^{2}E_{0}\left(t\right)}{\partial t_{i}\partial t_{j}}=\frac{\partial^{2}\langle \Psi_{0}\left(t\right)\left|H\left(t\right)\right|\Psi_{0}\left(t\right)\rangle }{\partial t_{i}\partial t_{j}}$$

evaluated at the symmetric configuration $t_{i}\equiv t$. In the space of the hopping parameters ($t\in R^{n}$) the latter is a stationary point w.r.t. any change orthogonal to the “breathing mode”, where each $t_{i}$ varies by the same amount ($t_{i}\equiv q$ for any *i*). This follows from the fact that the energy gradient at such symmetric configuration is obviously directed along such breathing mode. Hence, the eigenvalues of H but the one associated with the latter mode provide information about the intrinsic distortivity of the system.

It turns out that the Hessian is rather simple and can be computed by looking at a few distorted structures involving just one different hopping, e.g., $t_{1}$, the others being left unchanged ($t_{i}=t$ for i=2,…n). Indeed, thanks to the Hellmann–Feynmann theorem, for any $t\in R^{n}$, the energy gradient is a collection of ground-state one-body correlation functions,

$$\frac{\partial E_{0}}{\partial t_{i}}=-2\sum_{\sigma }ℜ\langle \Psi_{0}\left(t\right)\left|a_{i,,\sigma }^{†}a_{i+1,,\sigma }\right|\Psi_{0}\left(t\right)\rangle$$

where $t_{i}$ is used to describe the hopping between site *i* and site i+1, and periodic boundary conditions $a_{n+i}=a_{i}$ are implied. Any element of the Hessian above can be obtained from the first derivative of the correlations ${\langle a_{i,\sigma }^{†}a_{i+1,\sigma }\rangle }_{0}$ as functions of $t_{1}$ for fixed $t_{2}=t_{3}=\ldots =t_{n}=t$, when exploiting the rotational symmetry of the system ($H_{i+1,j+1}=H_{i,j}$). This symmetry further leads to explicit expressions for the eigenvectors of H and for the corresponding eigenvalues. Of interest here is the alternating mode, $t_{i}\propto {\left(-1\right)}^{n}$, and the corresponding eigenvalue

$$\kappa_{-}=\sum_{i=1}^{n}{\left(-1\right)}^{n}H_{1i}\equiv -4\sum_{i=1}^{n}\frac{\partial \langle a_{i,↑}^{†}a_{i+1,↑}\rangle }{\partial t_{1}}{|}_{t_{j}\equiv t}$$

**Figure 9:**
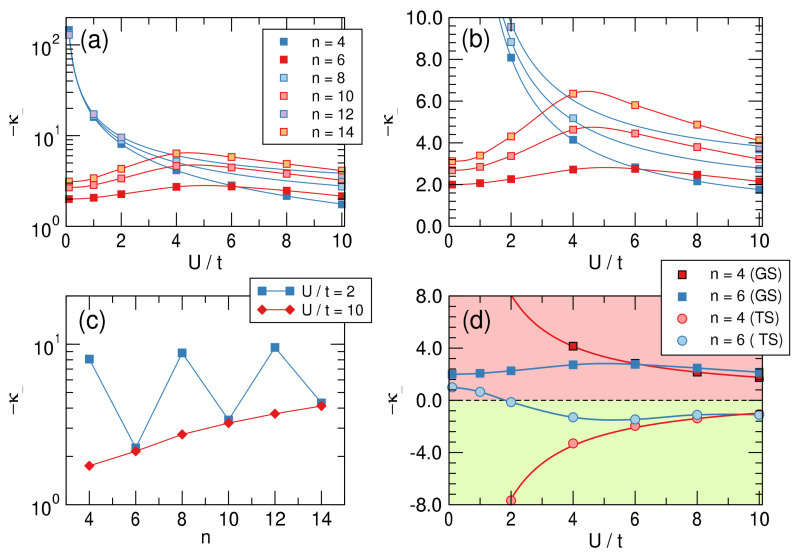
**Upper** panels: Intrinsic distortivities—$\kappa_{-}$ in a log-linear (**a**) and linear–linear (**b**) scale as functions of U/t for different values of *n*. (**c**) panel: Size-dependence of the distortivity in the weakly (U/t=2) and strongly (U/t=10) interacting limits. (**d**): Distortivity for the ground (GS) and the twin (TS) states for n=4,6. Notice that $\kappa_{-}>0$ (green colored area) means that the structure is stable against the bond alternation distortion.

In practice, we “distorted” the molecule by varying $t_{1}$ in the range 1±0.05, fitted the correlations $\langle a_{i,↑}^{†}a_{i+1,↑}\rangle$ to a third order polynomial to find the required derivatives, hence the “intrinsic” π-distortivity $\kappa_{-}$ for t=1. (The recipe can be easily translated into first quantization language, where $\langle a_{i,↑}^{†}a_{i+1,↑}\rangle$ becomes an element of the one-particle density matrix, in the basis of atomic states involved in the bonding.) The results of this analysis are reported in the top panels of Figure 9, both in a linear-log scale (left) and in a linear-linear one (right), and clearly show that aromatic molecules are distortive ($\kappa_{-}$ is always negative), although the distortivity of antiaromatic systems can be one to two orders of magnitude larger. The latter features a monotonic decrease of distortivity for increasing interaction strength U/t as a consequence of the general weakening of the bonds that occurs when increasing *U*. By contrast, in aromatic systems this “ordinary” behavior sets in only for sufficiently strong interactions and at first, for small values of U/t, $\kappa_{-}$ is seen to increase with interaction strength (in this context notice that U/t∼4 is an appropriate value for a carbon π system). This is clearly due to the effect that the interaction strength has on aromaticity, the key “protecting” factor that minimizes the π-distortivity.

The lower panels of Figure 9 provide further insights into π-distortivity. On the left we plot the distortivity as a function of the system size for two values of U/t on the opposite sides of the “bell” of the upper right panel. The graph makes clear that distortivity increases, on average, with increasing size, as evidenced from the red curve that refers to a strongly interacting situation. The latter can be easily extrapolated to the infinite size limit to give $\kappa_{-}∼4-5$, which is our estimate of the intrinsic “Peirls distortivity” for the given U/t=10. The same occurs in the weakly interacting limit, although here $\kappa_{-}$ alternates evidently between antiaromatic and aromatic species.

Finally, the right panel of Figure 9 shows the “dual” character of the so-called twin state, the excited singlet state that in a valence bond picture corresponds to an anti-bonding combination of Kekulé structures. Such a dual character is manifested in benzene by the remarkable frequency exaltation of the Kekulé (b${}_{2u}$) mode in S${}_{1}$ compared to S${}_{0}$ [62], which is suggestive of a stabilizing π system in S${}_{1}$, opposite to the ground-state [63]. The figure shows that if n=4 is strongly distortive in the ground-state it is stable (and stiff w.r.t. to bond alternation) in the twin-state and conversely for n=6, which is mildly distortive in the ground-state and becomes mildly stable in that excited state, provided U/t is not too small. Further insights into distortivity and aromaticity will be provided in a forthcoming article.

## 5. Conclusions

Multiple bonding is a striking structural motif, particularly in carbon chemistry. However, “C supremacy” has led over the years to misconceptions and wrong beliefs that only a thorough investigation of the last few decades could reveal. Multiply bonded structures are the result of a delicate interplay between opposing forces due to the σ and the π components; whether one is distortive or resisting depends on the kind of distortion.

All this being now well established, much effort is yet required in telling the correct story about π bonds to the next generation of chemists.

## Figures and Tables

**Table 1:** Buckling height (*h*) in some Silicene “dots” (Figure 4), as obtained with different density functionals and a 6-31++G${}^{**}$ basis set. In hexasilabenzene *h* was determined from the heights of the Si atoms above and below the natural plane, which is midway between the planes defined by up- and down-Si atoms. For Si${}_{10}$–naphatelene and Si${}_{54}$–circumcoronene *h* was defined similarly but at the center of the molecule only.

*h*/Å	Si${}_{6}$H${}_{6}$	Si${}_{10}$H${}_{8}$	Si${}_{54}$H${}_{18}$
HF	0.18	0.17	0.18
PBE	0.45	0.44	0.46
B3LYP	0.43	0.41	0.42
M06L	0.40	0.38	0.38
M06	0.48	0.49	0.51
M062X	0.37	0.40	0.42
M06HF	0.33	0.43	0.50

## Data Availability

The optimized geometries are made available as Supporting Information (Geometries.tgz).

## Supplementary Materials

The following are available online: file: Geometries.tgz.

## Sample Availability

Not available.

## Abbreviations

The following abbreviations are used in this manuscript:

DFT	Density Functional Theory
AO	Atomic Orbital
MO	Molecular Orbital
NBO	Natural Bond Orbital
BLA	Bond Length Alternation
AE	Atomization Energy
